# Uterus Transplantation with Live Donors: Screening Candidates in One French Center

**DOI:** 10.3390/jcm9062001

**Published:** 2020-06-25

**Authors:** Marie Carbonnel, Aurelie Revaux, Elena Menzhulina, Lea Karpel, Renaud Snanoudj, Morgan Le Guen, Dominique De Ziegler, Jean Marc Ayoubi

**Affiliations:** 1 Department of Obstetrics and Gynecology, Hospital Foch, University of Versailles, Saint-Quentin-en-Yvelines, 55, avenue de Paris, 78000 Versailles, France; aurelie.revaux@hopital-foch.com (A.R.); menzhulinaelena@hotmail.com (E.M.); l.karpel@hopital-foch.com (L.K.); ddziegler@me.com (D.D.Z.); jm.ayoubi@hopital-foch.com (J.M.A.); 2 Department of Nephrology and Transplantation, University of Versailles, Saint-Quentin-en-Yvelines, 55, avenue de Paris, 78000 Versailles, France; r.snanoudj@hopital-foch.com; 3 Department of Anesthesiology, Hospital Foch, University of Versailles, Saint-Quentin-en-Yvelines, 55, avenue de Paris, 78000 Versailles, France; m.leguen@hopital-foch.com

**Keywords:** uterine, uterus, transplant, transplantation, screening

## Abstract

We report our experience regarding the profile and screening process of potential recipients (R) and their live donors (D) in our Uterus transplantation (UTx) trial from 2014 to 2020. The initial screening was performed using medical questionnaires and consultations. The second step of the screening consisted of two individual interviews with an independent multidisciplinary committee. Then, a complete medical, biological and imaging assessment of the directed living D, the R, and her partner was performed over a two-day hospitalization. A total of 239 women contacted our department: 165 potentials R and 74 potentials D. During the first step of screening, 141 R and 45 D were excluded. Only 12 R/D pairs were pursued. During inclusion, 10 R/D pairs were excluded. One R/D pair is still under evaluation. Finally, only 1 R/D pair was definitively included (0.6%), which led us to perform the first French UTx in March 2019 with a successful graft. The primary limiting factors of inclusion were due to very strict criteria and difficulty of having a suitable directed living D. The International Society of UTx (ISUTx) guidelines based on worldwide results of trials can help ease our inclusion criteria in the future while remaining safe for patients.

## 1. Introduction

Absolute Uterine Factor infertility (AUFI) affects approximately 1 in 500 women [1]. It can either be congenital or acquired. Congenital AUFI is constituted by the Mayer-Rokitansky-Küster-Hauser (MRKH) syndrome whereby the uterus and a fraction of the vagina are congenitally absent, a condition encountered in 1/4500 female births [2]. Acquired AUFI are the cases of hysterectomies performed in young women of reproductive age for obstetrical hemorrhage or other causes such as, for example, early stage cervical cancer or unmanageable fibroids [3]. Relative uterine factor infertility (RUFI) results from conditions leading to severely non-functional uteruses such as intra uterine adhesions (Asherman syndrome). When the uterus is absent or non-functional, the only available option that allows the preservation of the genetic parenting has been, until recently, gestational surrogacy. This is, however, an expensive approach, which is illegal in several countries such as France (Law n° 94-653 of 29 July 1994). Since the report in 2014 of the first case of successful pregnancy in a transplanted uterus by Brannström’s team [4], more than 70 cases of uterine transplantation (UTx) have been performed across the world with 52 reported in peer reviewed journals [5,6,7,8,9,10,11,12,13,14,15,16,17]. More than 20 healthy children were born after UTx, with 16 published in peer reviewed journals, the majority from live donors (LD) [4,18,19,20] and three births from deceased donors (DD) [20,21,22]. UTx is the first time-limited organ transplantation performed not for a vital indication, but for improving the quality of life with potential life births. Some teams have published their data about their screening process [23,24,25,26,27]. In France, two teams received an authorization to conduct an UTx, the Limoges using DD (interrupted for administrative reasons) and our own team with the use of LD. We performed with success the first French case of UTx on March 2019. We report here the profile of the potential candidates in our clinical trial.

## 2. Materials and Methods

As part of the preinclusion phase of our UTx clinical trial (n° NCT03689842), we considered patients with AUFI who had independently contacted our institution. Starting in October 2014, we queried whether they would be interested in participating in our study. We collected data from this date until March 2020. Our trial is a prospective, open-label, non-randomized, single center study conducted in 10 patients with an MRKH syndrome or having suffered a hysterectomy (with no radiotherapy) for cervical cancer (stage IA FIGO 2018) in remission for ≥5 years, benefiting from a directed LD. There was no advertising or recruiting for the clinical trial. This study was approved by our institutional review board (CEROG 2018-GYN-1202) in January 2019.

### 2.1. First Screening Step

One hundred and sixty-five potential recipients interested in the trial contacted us with letters, dedicated email, or through direct consultation with our referring investigator. A medical heath history questionnaire was sent to all patients who didn’t have an appointment in order to evaluate if they could go further in the trial. We received 9 letters from potential recipients, and 64 patients contacted us only by mail. In total, 92 potential recipients and their partners had a consultation with our referring investigator (gynecologist). In addition, 45 potential donors had a consultation. The following data were collected from the potential recipient: age, residency, language, body mass index (BMI), cause of AUFI, directed donor, parental, marital, smoking and obstetrical status, medical and surgical history, medications, and partner’s opinion about UTx. In case of MRKH, any possible associated malformation and vaginal reconstruction were noted. The following data were collected for the directed donors: relationship status with the recipient, age, BMI, obstetrical history, smoking status, medical and surgical history, medications, and ABO compatibility with the recipient. If the recipient, the recipient’s partner, and their directed donor did not have exclusion criteria, they received medical information about the project. This included the experimental nature of the program, a description of the trial plan with all the pre-, intra and post-transplantation procedures, risks incurred, research advances in terms of transplantation, and the worldwide experience. Ultimately, the donors and recipients had a clinical check up with blood pressure, heart rate, abdominal, and gynecological examination conducted by the referring investigator.

### 2.2. Second Step of Screening

Donor, recipient, and their partner were interviewed individually after a 3-month reflection period by the Foch Hospital uterine transplantation multidisciplinary independent committee (composed of an anesthesiologist, a transplant surgeon, a gynecologist specialized in medically assisted procreation, and a psychologist/ psychiatrist specialized in transplantation) The donor/recipient pair was informed of the lack of financial cost, as the study sponsor would be responsible for all costs related to the surgical procedure, transport, housing, and loss of earning during work interruption. During the first interview, the consent form and the information note were given and explained to the donor, recipient, and their partners. They were informed by the committee of the procedure, risks of complications, or failure in each step of UTx. They had an individual interview with the psychologist to evaluate their ability to participate in the UTx trial and undue familial pressure, especially for donors. After the first and the second interview (following a 3-month reflection period), the multidisciplinary uterine transplantation committee decided on the patient’s eligibility. Finally, the consent dated and signed by the donor, the recipient and her partner were collected by the investigator after the approval of the multidisciplinary committee.

### 2.3. Inclusion

A medical, psychological, biological, and imaging assessment of the directed living donor, the recipient and her partner were performed during a 2-day hospitalization in order to confirm the eligibility.

#### 2.3.1. Step 1

During the first day, a general assessment (medical, laboratory and immunological assessment), anesthesia consultation, and psychological consultation were performed. Based on the results, the inclusion process could be stopped and the couple excluded.

#### 2.3.2. Step 2

During the second day, a general assessment (imaging) and a psychological consultation were performed. Depending on the results, the inclusion process could be stopped and the couple excluded. In case of inclusion, a donor was interviewed individually by the biomedicine agency Living Donor Committee. After the living donor committee approval, the donor had to express her consent to the president of the Court of first Instance.

#### 2.3.3. Definitive Inclusion

Obtaining 10 cryopreserved embryos at the blastocyst stage from the recipient and partners gametes was a requirement for including the recipient and their directed donor. Seven days before surgery, a shorter medical, psychological, biological, and imaging assessment was performed again in order to check the inclusion criteria. The screening and inclusion criteria are summarized in Table 1.

Quantitative data were expressed as a mean (range) and qualitative data were expressed as percentages.

## 3. Results

**Figure 1:**
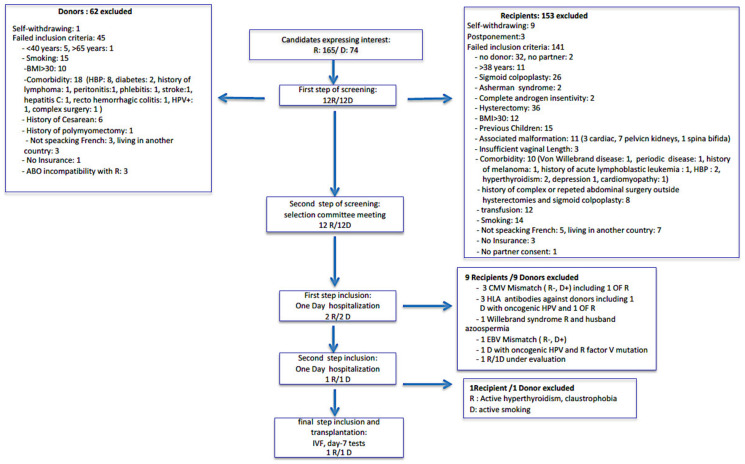
Results of screening and inclusion process for candidates. OF: ovarian failure.

A total of 239 women contacted our department for our UTx trial from October 2014 to March 2020: 165 potential recipients and 74 potential donors. The characteristics of potential recipients and donors are summarized in Table 2. One hundred and twenty-five potential recipients had one potential directed donor, and 8 two potential directed donors. We had two altruistic donor requests. We had detailed information for only 74 potential donors. In addition, 125 (76%) potential recipients had MRKH syndrome, 27 had associated malformation including 11 incompatibles with our trial, 26 recipients had a sigmoid colpoplasty, 3 had a too short vaginal length. Furthermore, 36 (21.8%) potential recipients had hysterectomies: 12 for malignancy (1 sarcoma, 1 choriocarcinoma, 2 ovarian cancer, 4 cervical cancer with radiotherapy) 15 for obstetric complications, 6 for benign disease (2 endometriosis, 4 myomas), and two missing data. Two potential recipients had complete androgens insensitivity and 2 Asherman syndrome. Thirteen potential recipients with hysterectomy already had biological children and two patients had an adopted child. No case of surrogacy was reported. In our trial, only directed donors were included. One sister was an identical twin of a MRHK patient. She was 34 years and couldn’t be included because of her young age. Results of initial screening and process for candidates are summarized in Figure 1. During step one of screening, among 165 recipients, we had 9 dropouts, 3 patients under reflection and 141 patients didn’t meet inclusion criteria. Among 74 donors, we had 1 dropout and 45 didn’t met the inclusion criteria. Concerning the recipients, the most frequent reason for exclusion were: absence of donor, hysterectomy (except for early stage of cervical cancer), associated malformations, previous children, sigmoid colpoplasty, age >38 years, BMI>30, smoking, history of transfusion, and comorbidity. For donors, the most frequent reasons for exclusion were smoking, obesity, and comorbidities. Several exclusion criteria could be found for the same patients. Only 12 recipients, their partners, and their directed donors met the selection committee in the second step of screening. They all were allowed by the committee to continue the process. During the first step of inclusion, 9 R/D were excluded: 3 R had CMV mismatch with D (R-, D+) including one with ovarian failure, 3 R had HLA antibodies against donors including 1 D with oncogenic HPV + and one R with ovarian failure, 1 R had EBV mismatch with D (R-, D+), 1 D had oncogenic HPV+ and R a Factor V mutation and 1 R had Willebrand syndrome with partner’s azoospermia. During the second step of inclusion, 1 R/D was excluded: R had hyperthyroidism and claustrophobia making MRI and CT-scan impossible and D didn’t stop smoking. One R/D pair is still under evaluation. Finally, a 1 R/D pair was definitively included thereby leading to perform the first French UTx in March 2019 with success of the graft.

## 4. Discussion

It is important to select the best R and D candidates in order to obtain a functional graft and, at term, a pregnancy with the birth of a healthy child with a minimum of risk for recipients, donors, and resulting children. Moreover, this is a new procedure still in the experimental stage and the precautionary principle must be widely applied. We found that, out of 165 UTx candidates with a directed donor, only one was able to integrate our trial; this is the lowest inclusion rate in reported trials [26,27]. We can query about the disparities in the number of inclusions in our trial and those of some other teams, and envision slightly loosening some of our inclusion criteria. Conversely, certain inclusion criteria could be stricter in the light of the recent data like the age of donors [6,7,15]. As in the case of the Baylor and German trials, most of our donors and recipient candidates were screened out during the noninvasive and cost-efficient initial screening [26,27]. We performed a multistep screening and inclusion process, including extensive psychological assessment.

In our trial, we only used LD directed to the recipients. These had emotional or genetic relationships with the R on the model of kidney transplantations. Most often, they were the mothers, as in the majority of UTx trials. In 72% of cases, the directed donor failed to fill the inclusion criteria at the first step of the screening. In order to increase the number of potential compatible donors, the Baylor’s team included altruistic donors [26]. Non-directed living organ donation is still controversial because of the potential risks of organ trading [28]. On the other hand, unlike the kidney or liver, the uterus at the time of donation is no longer of use for the donor. Patients who must have a hysterectomy for a medical reason but with a normal uterus could be good donors too [29]. This situation occurs in female-to-male transgenders; among them, 84% wanted to volunteer for donation in a survey [30]. Re-use of the uterus of recipients after closure hysterectomy could be attractive but is at greater risk of rejection [31]. Altruistic donation is cultural and more developed in the US, where a survey about public attitude toward vascularized composite allograft donation showed that 70% of women were willing to donate their uterus [32]. However, we received two potential altruistic donors in our trial. Mentalities may change, especially if the risks for the donor decrease [33]. Donor surgery is far more extensive than a simple hysterectomy in order to preserve vessels, which incurs risks of major complications, especially to the ureter [34]. In an attempt to reduce these complications and the duration of surgery, teams including ours are working on minimally invasive surgery and robotics [9,11,12,14,35,36,37] and the use of ovarian or uteri-ovarian veins instead of uterine veins for assuring the venous outflow in the recipient [12,14,38]. Testa et al. reported a live birth after laparotomy retrieval of the uterus using only the ovarian vein [38,39]. Further investigations are required to confirm that ovarian veins are sufficient for the uterine drainage, implantation, and normal pregnancy without complications. If proven efficient, this approach could greatly simplify the surgery for LD. Age and hormonal status of the donor could be major factors of success of the UTx. Outcomes of post-menopausal D were poorer in Czech, Swedish, and German trials [6,7,15]. Hormonal treatment always prescribed for few months in LD before UTx could improve the graft function and is necessary to evaluate endometrial thickness. Exclusion of D >60 years of age or menopausal for >5 years needs to be discussed. This suggests negative age-related changes in uterine vasculature. Contrast enhanced magnetic resonance imaging and CT, which were initially performed to evaluate vessels, did not visualize suboptimal arterial flow through uterine arteries with initial atherosclerotic lesions in the aborted German LD case [6]. Conventional arteriography was added in later LD trials including in ours, which seem necessary to evaluate the uterine artery status of LD [5]. Suboptimal quality of vessels could be a major reason of potential D exclusion [40]. History of at least one normal pregnancy, without recurrent miscarriages seems to be a reasonable precaution for LD. We could however include D with the history of one Cesarean section as good pregnancy issues have been reported in these cases [19,39]. Younger age of inclusion is variable among the trials. They should in principle be past their childbearing years. Some altruistic donors were much younger: 34, 26, and 42 years in Baylor’s trial [13]. Including donors under 40 years of age mandates an extensive psychological evaluation to be certain that the LD will not change her mind, which leads to irreversible infertility.

The risk of infection is high mainly during the first six months after a solid organ transplantation because of the immunosuppressive therapy and during pregnancy due to the physiologic immunomodulation. Explantations of the grafted uterus for uterine abscess, HSV, and candida infections have been reported [7,15,17]. A great amount of attention should be attributed to risk of infection, and optimal prevention should be undertaken in D and R. We were probably right to avoid CMV-positive donor grafts into CMV-negative recipients, considering the potential deleterious transfection and pregnancy. This situation (D+, R−) is unfortunately frequent because D who are older and have given birth are often positive, while R being younger and without children are more likely to be negative. Cervical CIN2 has been reported in R after UTx without any previous dysplasia or HPV in D [41]. Then, it is clear that HPV-included lesions in both D and R should preclude transplantation and HPV vaccine in R should be encouraged before UTx. ABO incompatibility is around 36% for kidney transplantation [42]. As an HLA mismatch, it is a major reason for exclusion.

UTx from deceased donors, still in its infancy, is an interesting alternative to avoid LD complications [21,43]. It could facilitate the anastomosis in the recipient with larger vessels (iliac vessels). Only eight cases have been reported so far, with lower success than LD but a lot of teams are performing trials [7,16,17,21], and three children have been described after UTx from DD, which proves its feasibility [20,21,22]. Other limitations exist for DD like low availability, short time to evaluate the graft, or longer ischemia time. DD could be another option for R without compatible LD. LD and DD could both be options in the future as for other solid organs’ transplants.

Up to now, R were mostly MRKH, the congenital AUFI associated with shortened vagina. A sufficient vaginal length is necessary to perform a good anastomosis with the graft and decrease the risk of vaginal stenosis after UTx [7]. Vaginal reconstructions with intestine segment could lead to poor results for pregnancy probably due to inflammation. The only one R in whom this type of reconstruction had been conducted suffered recurrent miscarriages without any childbirth [44]. They were often performed until recently and we had to exclude 20% of the potential MRKH patients for this reason. Self-dilatation should be recommended [45] for all MRKH patients with vagina atrophy. MRKH is often associated with unique kidneys: some were included and obtained healthy children but had more preeclampsia [19] and renal toxicity, which is more frequent with immunosuppressive therapy. An increased monitoring should be performed for them. Presence of a pelvic kidney was excluded in our trial because of possible surgery complications for vascular anastomosis of the graft to external iliac vessels. A Swedish team included some cases without any complications [15]. AUFI due to hysterectomy could represent a huge number of R, more than 60% of candidates as suggested in different teams [23,25,26]. Surgery in the R could be more complex because of adhesions, which explain why this kind of R was smaller in first trials. Particular caution should be taken for patients with a history of cervical cancer because of the risk of recurrence. Only the early stages without radiotherapy and no sign of recurrence for ≥5 years should be considered, as in the Swedish series [15]. No data are available for other kinds of cancer. History of hysterectomy for benign pathology could be more acceptable. Two cases of hysterectomy for myomas have been reported [13] and one case of post-partum hysterectomy for hemorrhage [8]. Transfusions and history of previous pregnancy increase, however, the risks of rejection. The fact that the R already has children should be discussed as a possible exclusion factor because of the current risks involved in UTx [46], but, in the Baylor trial, more than 50% of interested potential recipients had children [26]. Finally, RUFI could be included. Indeed, in some cases, pregnancy is impossible and multiple ART attempts have failed. One case of Asherman syndrome was included in the Indian trial [11]. The total incidence of AUFI and RUFI is not known but could involve more than 150,000 women during childbearing age in Europe [1]. Even if it is not possible to fulfill every patient’s dream of motherhood, the number of UTx performed is bound to increase. The Montreal criteria for the ethical feasibility of UTx revised in 2013 are helpful to define the indications and limits [47]: Importantly, the first condition for R is to be a genetic female of reproductive age.

A sufficient but thoughtful screening process of living donors and recipients is essential and should aim both to assure donor/recipient safety and to provide good quality grafts. The risk/benefit ratio and evolutions of UTx will be determinant to define which R and D could be included in the future.

## Figures and Tables

**Table 1:** Screening and inclusion criteria.

	Recipients	Donors
**Screening criteria step 1**	Aged 18–38 years Presenting with directed donors BMI < 30 MRKH type 1 Or MRKH type 2 with unique orthotopic kidney Or after hysterectomy for cervical cancer stage IA (FIGO 2018) remission, 5 years no radiation therapy No children In stable partnership No treatment ABO group Compatibility with the donor Residual vagina 6 cm or more beneficiary of a social security No severe comorbidities No associated malformations No azoospermia in the partner No history of cancer (except cervical cancer) No history of transfusion No history of complex abdominal or pelvic surgery No sigmoid colpoplasty No transmissible infectious diseases in the couple No smoking, no history of active drug abuse or alcoholism Couple speaking French.	Aged 40–65 years Directed with recipient BMI < 30 Having at least one child in good health (no recurrent miscarriages, no premature deliveries, no preeclampsia, no Caesarian) ABO group Compatibility with the donor beneficiary of a social security No severe comorbidities No history of cancer No uterine scarring and major uterine surgery No history of conization No uterine disease No transmissible infectious diseases No History of major abdominal or pelvic surgery No smoking, no history of active drug abuse or alcoholism Speaking French.
**Screening criteria step 2**	Favorable psychological assessment Recipient and partner having given their informed consent to participate in the study Multidisciplinary comity agreement	Favorable psychological assessment Donor having given her informed consent to participate in the study Multidisciplinary comity agreement
**Inclusion criterias step 1**	Normal thrombophilia tests HLA compatibility with donor Negative HPV oncogene test Negative PCR herpes simplex viruses 1 and 2 test No gynecologic infection No transmissible infectious diseases in the couple Good ovarian reserve: AMH > 1.5, AFC > 10 Normal pelvic ultrasound No diabetes Good renal, hepatic function Normal hemostasis Normal spermogram for the partner	Normal vaginal smear HLA compatibility with recipient Negative HPV oncogene test Negative PCR herpes simplex viruses 1 and 2 test No gynecologic infection No transmissible infectious diseases Positive CMV and/or EBV serologies (if the recipient is negative) Positive HHV8 serology (if the recipient is negative) Normal pelvic ultrasound No diabetes Good renal, hepatic function Normal hemostasis Normal endometrial biopsy
**Inclusion criteria step 2**	Normal echocardiogram Normal abdominal and pelvis MRI Normal Chest X-ray	Good quality uterine vessels as examined by angio-MRI, angio-CT scan and arteriography Normal abdominal and pelvis MRI Normal echocardiogram Normal neck vessels sonography Normal mammogram Normal abdominal ultrasound Normal Chest X-ray
**Definitive inclusion**	At least 10 frozen embryos with IVF Normal tests 7 days before surgery	Normal tests 7 days before surgery

**Table 2:** Characteristics of potential recipients and donors.

			Recipients *n* (%) or Mean (Range)	Donors *n* (%) or Mean (Range)
			165	74
Cause of AUFI	MRKH S		125 (75.8)	N/A
	Associated M		27(16.3)	
		Cardiac	3	
		Unique Kidney	15	
		Unique pelvic Kidney	2	
		Pelvic Kidney	7	
		Spina bifida + bone M	1	
		hearing M	1	
	Vaginoplasty	Sigmoid colpoplasty	26	
		Others	24	
		Missing data	7	
	Hysterectomy		36 (21.8)	
		Benign disease	7	
		Malignancy	9	
		Obstetric complications	15	
		Missing data	5	
	Asherman		2 (1.2)	
	Syndrome			
	Complete androgene Insensitivity		2 (1.2)	
Residency		Paris and around	51(30.9)	18 (24.3)
		Other areas in France	101 (61.2)	48 (64.9)
		Foreign countries	7(4.2)	4 (5.4)
		Missing data	6 (3.6)	4 (5.4)
French speaking		Yes	152 (92.1)	67 (90.5)
		No	5 (3)	3 (4)
		Missing data	8 (4.8)	4 (5.4)
BMI (kg/m^2^)			23.9 (17.3–41.7)	25.1(17.7–37.2)
Age (y)			30(18–46)	50.7 (28–65)
Smoking status		Yes	14 (8.48)	19 (25.7)
		No	143 (86.7)	58 (29.1)
		Missing data	8 (4.8)	7 (4.2)
Committed relationship/married			154 (93.3)	N/A
Single			1 (0)	
Missing data			10 (6)	
Prior children		Yes	15 (9)	63 (85.1)
		No	127 (77)	2 (2.7)
		Biological	13 (7.8)	63 (91.1)
		Adopted	2 (1.2)	0 (0)
		Missing data	8 (4.8)	9 (12.1)
Donor Relationship with Recipient		Mother		45 (60.8)
		Friend		3 (1.8)
		Sister		6 (3.6)
		Sister in law		2 (2.7)
		Mother in law		3 (1.8)
		Cousin		3(1.8)
		Aunt		1 (1.3)
		Altruistic		2 (2.7)
		Missing data		9 (12.1)
Hormonal status		Premenopausal	149(90.3)	30(40.5)
		Postmenopausal	0 (0)	37 (50)
		No ovaries	5 (3)	1 (1.3)
		Androgen insentivity	2 (1.2)	0
		Missing data	9 (5.4)	6 (8.1)

M: malformation, N/A: non applicable, S: Syndrome
